# Lost Work Opportunity Score Predicts Health in Retirement

**DOI:** 10.1093/geroni/igab046.2183

**Published:** 2021-12-17

**Authors:** Maren Wright Voss, Man Hung, lorie Richards, Wei Li, Pollie Price, Alexandra Terrill, Lori Wadsworth

**Affiliations:** 1 Utah State University, Salt Lake City, Utah, United States; 2 Roseman University, South Jordan, Utah, United States; 3 University of Utah, Salt Lake City, Utah, United States; 4 Brigham Young University, Provo, Utah, United States

## Abstract

Objectives: Under-reporting of unemployment or forced retirement has consequences for measuring the impacts of job changes on health at retirement. We analyzed a comprehensive three-part measure of lost work opportunity for evidence of impact on health.

Methods: We combined variables from the Health and Retirement Study for 2,576 respondents assessing unemployment, forced retirement, and earlier than planned retirement into a single lost work opportunity score (LOS). We evaluated the reliability and unidimensionality of the LOS. We conducted multivariate regression to assess health impacts controlling for age, gender, education, race, ethnicity, and prior health status.

Results: The Cronbach’s Alpha for the LOS was a = 0.76 and the LOS variables primarily loaded onto a single component demonstrating undimensionality. The LOS significantly predicted self-reported health (□= .16; p < .001) with higher lost work associated with negative health outcomes (Cox and Snell R2 = 0.07). The LOS score significantly predicted mental health declines (□ = .07; p = .002)(Cox and Snell R2 = 0.07).

Discussion: Population-level data indicates that health declines following both unemployment and retirement, but there is ample evidence that early or planned retirements do not show the same negative health impacts. We examined the health impact of retirement using the construct of lost work opportunity rather than voluntary or involuntary retirement, per se. Our findings indicate that as much as 7% of negative health changes in the early retirement years could be attributable to employment changes that were unplanned or experienced as outside the retiree's control.

